# Dynamics and ecological reassembly of the human gut microbiome and the host metabolome in response to prolonged fasting

**DOI:** 10.3389/fmicb.2023.1265425

**Published:** 2023-10-03

**Authors:** Xiaopu Sang, Shenghui Li, Ruochun Guo, Qiulong Yan, Changxi Liu, Yue Zhang, Qingbo Lv, Lili Wu, Jie Ma, Wei You, Ling Feng, Wen Sun

**Affiliations:** ^1^School of Life Science, Beijing University of Chinese Medicine, Beijing, China; ^2^Puensum Genetech Institute, Wuhan, China; ^3^Department of Microbiology, College of Basic Medical Sciences, Dalian Medical University, Dalian, China; ^4^School of Traditional Chinese Medicine, Beijing University of Chinese Medicine, Beijing, China; ^5^Key Laboratory of Health Cultivation of the Ministry of Education, Beijing University of Chinese Medicine, Beijing, China; ^6^Beijing Key Laboratory of Health Cultivation, Beijing University of Chinese Medicine, Beijing, China; ^7^Beijing Hospital of Traditional Chinese Medicine, Capital Medical University, Beijing, China; ^8^Second Affiliated Hospital, Heilongjiang University of Chinese Medicine, Harbin, China

**Keywords:** obesity, gut microbiome, metabolome, fasting, dynamics, ecological reassembly

## Abstract

**Introduction:**

Prolonged fasting is an intervention approach with potential benefits for individuals with obesity or metabolic disorders. Changes in gut microbiota during and after fasting may also have significant effects on the human body.

**Methods:**

Here we conducted a 7-days medically supervised water-only fasting for 46 obese volunteers and characterized their gut microbiota based on whole-metagenome sequencing of feces at five timepoints.

**Results:**

Substantial changes in the gut microbial diversity and composition were observed during fasting, with rapid restoration after fasting. The ecological pattern of the microbiota was also reassembled during fasting, reflecting the reduced metabolic capacity of diet-derived carbohydrates, while other metabolic abilities such as degradation of glycoproteins, amino acids, lipids, and organic acid metabolism, were enhanced. We identified a group of species that responded significantly to fasting, including 130 fasting-resistant (consisting of a variety of members of Bacteroidetes, Proteobacteria, and Fusobacteria) and 140 fasting-sensitive bacteria (mainly consisting of Firmicutes members). Functional comparison of the fasting-responded bacteria untangled the associations of taxon-specific functions (e.g., pentose phosphate pathway modules, glycosaminoglycan degradation, and folate biosynthesis) with fasting. Furthermore, we found that the serum and urine metabolomes of individuals were also substantially changed across the fasting procedure, and particularly, these changes were largely affected by the fasting-responded bacteria in the gut microbiota.

**Discussion:**

Overall, our findings delineated the patterns of gut microbiota alterations under prolonged fasting, which will boost future mechanistic and clinical intervention studies.

## Introduction

Diet intervention is considered an effective method to improve lifespan and slow the progression of various diseases (Carter et al., 2018; Mardinoglu et al., 2018; Tang et al., 2023). Common forms of intervention include caloric restriction (Barquissau et al., 2018), carbohydrate restriction (Harvie et al., 2013), ketogenic diet (Abbasi, 2018), and fasting (Aon et al., 2020; Horne et al., 2020). Caloric and carbohydrate restrictions had been shown to have metabolic benefits, such as reducing body fat and insulin resistance and inducing short-term improvement in plasma glucose and cardiovascular risk (Barquissau et al., 2018; Van Zuuren et al., 2018; Diaz-Ruiz et al., 2021; Mishra et al., 2021), while the ketogenic diet had been proven to decrease triglycerides in the liver and insulin resistance level in individuals with nonalcoholic fatty liver disease (NAFLD) (Luukkonen et al., 2020). As an “unusual” intervention method, fasting had also shown health benefits. Early studies had revealed that intermittent fasting can enhance the antioxidative metabolism and improve neurological symptoms through effects on certain gut microbial taxa in mouse models (Cignarella et al., 2018; Liu et al., 2020). In humans, intermittent fasting can activate white adipose browning to ameliorate obesity (Li et al., 2017), inhibit the proliferation of colorectal cancer (Weng et al., 2020), and improve metabolic abnormalities and healthspan (Etemadifar et al., 2016; Mattson et al., 2017; De Cabo and Mattson, 2019). Also, prolonged fasting (that is, food deprivation for over 2 days) can decrease disease activity in patients with rheumatoid arthritis (RA) (Hafstrom et al., 1988), improved insulin release and maintained glucose tolerance (Solianik et al., 2023; Tripolt et al., 2023), and has potential mood-enhancing and pain-relieving effects in chronic pain patients (Michalsen, 2010). Many of these studies also underscored that further evidence was essential for reducing safety concerns and more accurately defining the impact mechanism of various diet interventions, especially fasting, on human physiology.

In recent studies, increasing evidence indicated that dietary changes are able to alter gut microbial composition and function with profound effects on human health. For example, metabolic patterns of gut microbiota may change in response to seasonal dietary changes of the Hadza of Tanzania (Smits et al., 2017). Gut microbial composition and metabolite profiles can rapidly change to adapt to plant- and animal-based diets (David et al., 2014). Reduced carbohydrate consumption resulted in increased bacterial folate production, which may partially improve multiple metabolic factors, neutralize oxidative stress, and reduce inflammation in patients with NAFLD (Mardinoglu et al., 2018). Food, gut microbiota, and host are the main components of the gut ecosystem, which constitute a diet-microbiome-host interaction network. Previous studies often revealed the associations between the specific dietary substrate and gut microbial taxa or specific host factors, however, the direct interactions between microbiota and host are still underexplored. Human and their gut microbiota are considered as a holobiont and have co-evolved into a diverse and complex system for millions of years (Van de Guchte et al., 2018). A normal gut microbial ecosystem is dynamically balanced, with different bacteria having different niches within the environment. Some bacteria are efficient at breaking down food substrates and providing energy productions [e.g., short-chain fatty acids (SCFAs)] for other bacteria or host cells. Some bacteria can utilize other bacteria or host-derived substances, and these bacteria are most likely food-independent. The dynamic balance of the gut microbiota is considered to be an important factor in human health, and when this balance is disrupted (usually name as “gut dysbiosis”), it may further cause or aggravate a variety of systemic diseases such as inflammatory bowel disease (IBD) (Tomasello et al., 2016; Federici et al., 2022), colorectal cancer (CRC) (Zou et al., 2018), and autoimmune diseases (Kinashi and Hase, 2021; Liang et al., 2022).

Early studies had revealed that intermittent fasting or caloric restriction can enhance the antioxidative metabolism, improve neurological symptoms, and promote anti-tumor immunity through effects on certain gut microbial taxa in mouse models (Cignarella et al., 2018; Liu et al., 2020; Mao et al., 2023). Some studies have also explored the impact of gut microbiota after dietary restrictions on human health (Cignarella et al., 2018; Mardinoglu et al., 2018; Maifeld et al., 2021; Von Schwartzenberg et al., 2021). A randomized human intervention study demonstrated that when gut microbiota from individuals who underwent a very-low-calorie diet intervention were transplanted into mice, the mice experienced a reduction in body weight and adiposity compared to those that had received microbiota before the intervention (Von Schwartzenberg et al., 2021). In a study for obese people with NAFLD, a short-term low-carbohydrate intervention rapidly altered the composition of the gut microbiota (Mardinoglu et al., 2018). This change was accompanied by an enhancement in the folate production of gut microbiota and an increase in folate concentration in the host’s blood. This may partly explain how such an intervention improves host lipid metabolism, balances oxidative stress, and reduces inflammation. Overall, although there have been many studies examining the regulatory impact of the gut microbiota on host health during dietary restrictions, most of these findings are derived from research conducted on mice (Cignarella et al., 2018; Liu et al., 2020; Mao et al., 2023). However, it is well-known that there are substantial differences between the gut microbiome of mice and human, and many of these findings require further validation. Currently, there have been only a limited number of studies investigating changes in the gut microbiota in response to dietary restrictions in human. There is still a great deal to explore concerning the complex interaction between the gut microbiota, dietary restriction, and human health.

Based on these backgrounds, we thought that prolonged fasting is a unique model for investigating the direct interaction between gut microbiota and host: when there is no food available, the gut microbiota and host form into a transient new ecosystem. In this study, we analyzed the dynamic and ecological variability of gut microbiota of 46 obese individuals undergoing a 7-days medically supervised water-only fasting procedure combined with specific exercises (collectively called *bigu* in traditional Chinese medicine). Fecal, serum and urine samples were collected from the individuals before fasting, on the 3rd and 7th day during fasting, and on the 7th and 14th day after fasting, and were used for multi-omics analysis by both metagenome and metabolome techniques. These longitudinal datasets were able to identify survival strategies of gut microbiota under extreme nutritional deficiency based on a genome-centric strategy, and to interpret the microbiota-human inter-associations in this transient gut ecosystem.

## Results and discussion

### Study design, participants, and clinical characteristics

**Figure 1:**
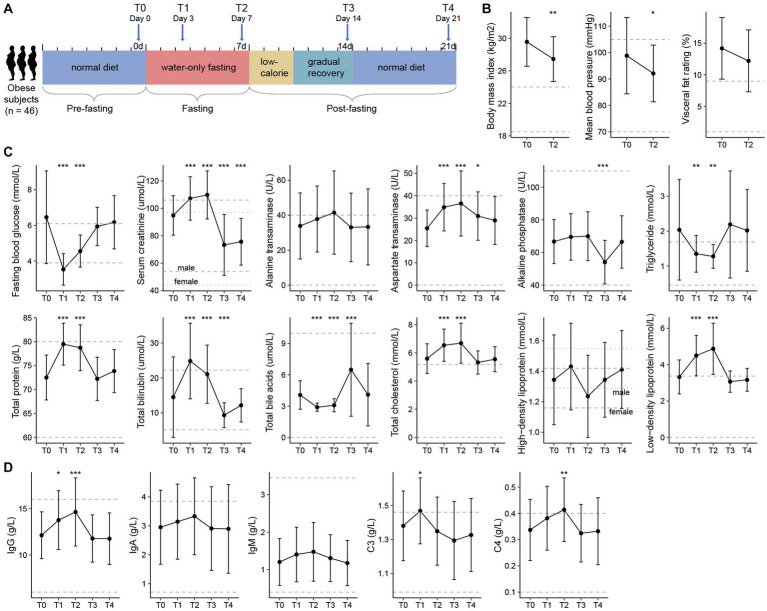
Experimental design and clinical characteristics of the participants. **(A)** Flow diagram of the clinical study. Forty-six obese volunteers who met the inclusion criteria were recruited and had finished the whole fasting procedure, including the pre-fasting stage (Day −6 to Day 0), fasting stage (Day 1 to Day 7), and post-fasting stage (Day 8 to Day 21). Clinical data and fecal samples were collected at five timepoints: T0 (Day 0, *n* = 46), T1 (Day 3, *n* = 46), T2 (Day 7, *n* = 46), T3 (Day 14, *n* = 45), and T4 (Day 21, *n* = 44). **(B)** Changes in the body mass index (BMI), visceral fat rating, and mean blood pressure of participants between T0 and T2. **(C)** Changes in the clinical parameters (i.e., routine blood, liver, and renal function parameters) of the participants at five timepoints. **(D)** Changes in the immune indexes of the participants at five timepoints. For **(B–D)**, filled dots represent the average value of the parameters in all individuals, and bars denote the lowest and highest values within 1.5 times the range of the first and third quartiles, respectively. Dotted horizontal lines represent the clinical typical ranges for each parameter. Significance levels compared with samples at T0 (Student’s *t*-test): *, *p* < 0.05; **, *p* < 0.01; ***, *p* < 0.001.

This study included 46 obese volunteers who participated in a 7-days medically supervised water-only fasting procedure and the subsequent recovery phase (3-days low-calorie diet and 4-days gradually recover to normal diet), with 7-days follow-up (Figure 1A). Fasting led to significant decreases in body weight (body mass index, from 29.6 to 27.5 kg/m^2^, *p* < 0.001), waist circumference (from 101.1 ± 10.3 cm to 96.1 ± 10.0 cm, *p* < 0.001), visceral fat rating (from 14.2 to 12.2%, *p* < 0.001), and mean blood pressure (from 98.8 to 92.1 mmHg, *p* < 0.001), along with a considerable decrease of visceral fat rate and metabolic rate (Figure 1B; Supplementary Table S1). Psychological evaluation during fasting revealed that the proportion of struggling participants decreased gradually from the first 3 days (71.4%) to the last day (21.4%), which effectively enhanced their confidence to complete the 7-days fasting procedure (Supplementary Figure S1A).

To further investigate the physiological variation of the volunteers during the whole experimental procedure, we collected the blood and urine specimens from each individual at five timepoints: T0 (*n* = 46), the day before fasting; T1 (*n* = 46) and T2 (*n* = 46), the 3rd and 7th day of fasting; and T3 (*n* = 45) and T4 (*n* = 44), the 7th and 14th day after fasting. Overall, the routine blood, liver, and renal function parameters of the participants had largely changed during the fasting period, however, most of these parameters returned to the original levels at the T3 and T4 timepoints (Supplementary Table S1). Individuals’ aspartate transaminase (AST), alkaline phosphatase (ALP), total bilirubin (TBIL), and total bile acids (TBA) deviated from the baseline at T3, but recovered at T4; in particular, their serum creatinine level was significantly decreased at both T3 and T4 (Figure 1C). Similarly, the immune indexes of individuals had been disturbed during fasting and showed no obvious abnormalities at timepoints T3 and T4 (Figure 1D). These results are in agreement with previous studies suggesting the safety of prolonged fasting for humans under careful care (Jiang et al., 2021; Tang et al., 2021), however, the potential benefits of fasting need to be studied further.

### Dynamics of the gut microbiota across the fasting procedure

The stool samples of 46 volunteers were collected at the same timepoints (T0-T4; Figure 1A) with their blood and urine specimens. To characterize the dynamics of gut microbiota in response to prolonged fasting, we performed whole-metagenome shotgun sequencing of fecal samples (representing 1,346 Gbp data; Supplementary Table S2) and carried out a metagenomic assembly of single samples and a co-assembly of samples for every individual at five timepoints. A total of 5,263 high-quality metagenomic-assembled genomes (MAGs) (≥90% complete and ≤ 5% contamination) were produced from the assembled contigs, which could be organized into 433 non-redundant clusters (referring to as “species” in the later text) for the species-level description of the gut microbiome (Jain et al., 2018; Supplementary Tables S3, S4). These species distributed a diverse range of bacterial phyla including Bacteroidetes (*n* = 76), Firmicutes (*n* = 204), Proteobacteria (*n* = 30), Actinobacteria (*n* = 18), Fusobacteria (*n* = 5), Verrucomicrobia (*n* = 2) and Synergistetes (*n* = 1), and an archaeal Euryarchaeota genome. Phylogenetic analysis showed that these species covered the major lineages of human gut microbiota (Supplementary Figure S2); especially, a majority of the species (55.4%) were currently uncultured. In addition, these non-redundant species achieved an average read mapping rate of 83.6% for the original fecal metagenomes (Supplementary Table S2), suggesting their effective representativeness of the gut microbiome.

**Figure 2:**
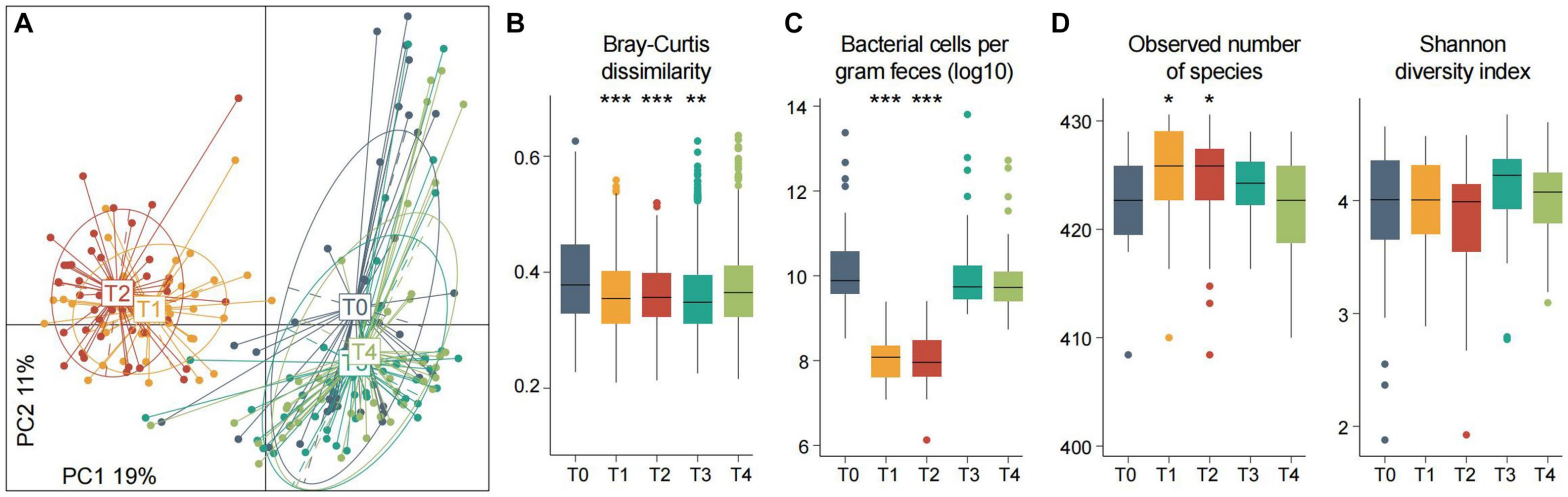
Gut microbiota composition and diversity at different timepoints of fasting. **(A)** Principal coordinates analysis (PCoA) of the Bray–Curtis dissimilarity at the species level of fecal samples. Samples are shown at the first and second principal coordinates (PC1 and PC2), and the ratio of variance contributed by these two PCs is shown. Lines connect samples that belonged to the same timepoint, and ellipsoids represent a 95% confidence interval surrounding each timepoint. **(B–D)** Changes of the between-sample Bray–Curtis dissimilarity **(B)** estimated bacterial cell numbers **(C)** and within-sample diversity **(D)** of the participants at five timepoints. Boxes represent the interquartile range between the first and third quartiles and the median (internal line). Whiskers denote the lowest and highest values within 1.5 times the range of the first and third quartiles, respectively; dots represent outlier samples beyond the whiskers. Significance levels compared with samples at T0 (Wilcoxon rank-sum test): *, *p* < 0.05; **, *p* < 0.01; ***, *p* < 0.001.

Using principal coordinates analysis (PCoA) of the gut species profiles, we observed a dramatic alteration of the overall microbial community structure across the experimental procedure (Figure 2A). This change immediately occurred during fasting began (from T0 to T1: *adonis R*^2^ = 18.3%, *p* < 0.001), with a similar tendency across all individuals. Inversely, the degree of gut microbiota alteration from 3rd to 7th fasting was little (T1 vs. T2: *adonis R*^2^ = 2.1%, *p* = 0.018). When start taking food again, the gut microbiota rapidly recovered, and particularly, the degree of gut microbiota alteration among T0, T3, and T4 was comparatively small (*adonis* R^2^ = 2.4%, *p* = 0.006). In addition, the dissimilarity of individuals’ gut microbiota during fasting was significantly reduced compared with those at the baseline or after fasting (Figure 2B), in agreement with the observation in PCoA analysis and suggesting that the pattern of gut microbiota alteration may be similar across different individuals.

We estimated the total amount of micrograms in feces using a qPCR analysis of the universal 16S rRNA primers. Consistent with the above findings, this analysis revealed that fasting led to a substantial reduction of bacterial amount (median cells per gram of feces, T_0/3/4_ vs. T_1/2_ = 6.0×10^9^ vs. 1.1×10^8^, *p* = 1.7×10^−20^), while the bacterial populations among T0, T3, and T4 have not differed (pairwise *p* > 0.05; Figure 2C). Some previous studies had shown that the use of antibiotics eliminated a large number of bacteria and led to a substantial reduction of the microbial diversity of gut microbiota (Chng et al., 2020). Unlike this, we found that both species richness and diversity indexes of the gut microbiota during fasting were approximate to or larger than those at baseline (Figure 2D). A similar increase was also observed in mice practicing intermittent fasting and in overweight women following low-calorie diets (Cignarella et al., 2018; Liu et al., 2020; Von Schwartzenberg et al., 2021). These results suggested that a considerable number of species still survived in the human gut under extreme nutritional deficiency. It should be noted that an increase in the microbiome diversity is generally considered a positive development. This is because higher diversity is often linked to improved health and immune system function, in contrast to lower diversity (Zheng et al., 2020).

### Overview of gut microbial functions and ecological patterns during fasting

**Figure 3:**
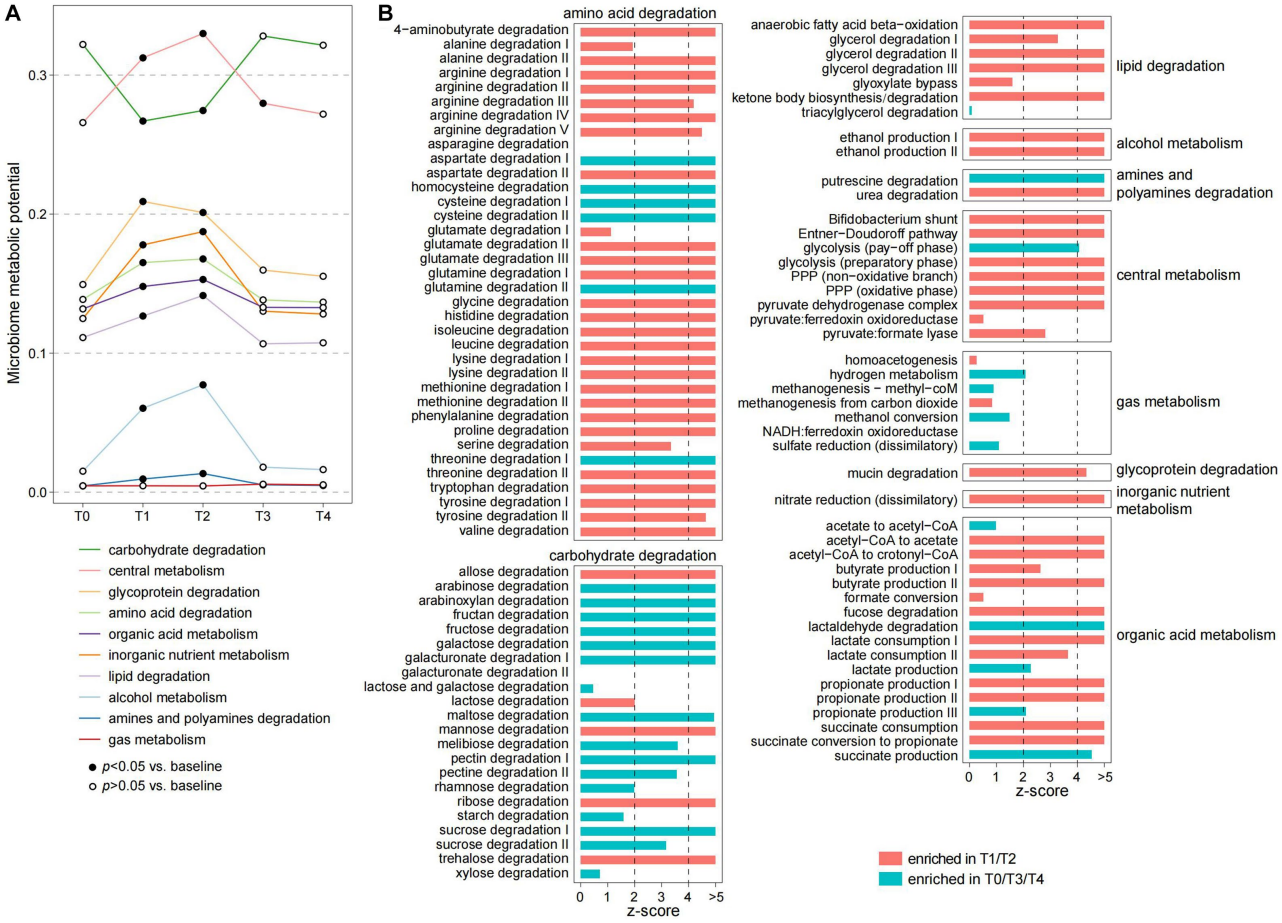
Alteration of the ecological pattern of gut microbiota during fasting. **(A)** Changes in the microbiome metabolic potential of the participants at five timepoints. Metabolic potentials of the gut microbiome are evaluated using the gut metabolic module (GMM) framework and grouped into 10 GMM categories. Dots represent the average value of each GMM category in all individuals, and the fitted dots denote a significance level of *p* < 0.05 (Wilcoxon rank-sum test) compared with samples at T0. **(B)** Changes of each GMM during fasting. Barplots indicate the z-score (Wilcoxon rank-sum test) of the comparisons of samples between T1/T2 and T0/T3/T4.

To investigate the functional dynamics of gut microbiota, we profiled the microbial functions of each sample via the KEGG (The Kyoto Encyclopedia of Genes and Genomes) and Carbohydrate-active enzymes database (CAZy) databases using a genome-centric approach method (Supplementary Table S4). Similar to the phylogenetic composition, PCoA analysis also supported that the functional microbiome is substantially changed during the fasting period and rapidly recovered at the T3 and T4 timepoints (Supplementary Figure S3). We then used the gut metabolic module (GMM) framework (Vieira-Silva et al., 2016) to evaluate the ecological properties of the gut microbiomes during and after fasting. Each GMM is defined as a set of orthologue groups that represent an enzyme-mediated ecological process, such as the degradation of a specific carbohydrate or amino acid, and the metabolism of an active small molecule substance (Vieira-Silva et al., 2016). During the fasting period, the individuals’ gut microbiome had remarkedly reduced in the capacity of carbohydrate degradation (Figure 3A). This reduction involved almost all plant-derived polysaccharides, including sucrose, starch, rhamnose, pectin, maltose, fructose, fructan, and arabinoxylan (Figure 3B), which appears to be a direct result of the lack of food. Inversely, almost all other GMMs involved in central metabolism, degradation of glycoproteins, amino acids, lipids, and organic acid metabolism were significantly enhanced in the gut microbiomes during fasting (Figures 3A,B).

The enhancement of central metabolism, especially the pentose phosphate pathway (PPP), during fasting was a typical phenomenon. PPP, a pathway parallel to glycolysis that is involved in the direct oxidation of glucose (Horecker, 2002), is important for generating NADPH, which is an essential cofactor for glutathione- and thioredoxin-dependent enzymes that defend cells against oxidative damage. Since the oxidative stress of the gut environment is profoundly increased during fasting, this pathway might be important for protecting bacteria from oxidative stress. Another noteworthy finding was that, unlike the plant-derived polysaccharides, the degradation of several animal-derived carbohydrates such as lactose and ribose was increased during fasting (Figure 3B). This finding is similar to the metabolic characteristics of gut microbiota of animals during hibernation (Carey et al., 2013; Sommer et al., 2016), suggesting that gut bacteria may be involved in metabolizing host-derived substances. Likewise, the metabolism of mucin, a glycoprotein component of the host intestinal mucosa, was increased during fasting. Similarly, the gut bacteria might also use certain small molecules, such as ethanol, nitrogen, and short-chain fatty acids (i.e., acetate, propionate, and butyrate), as energy sources during fasting, leading to a significant enhancement of the metabolic capacity of these substances. Overall, our findings suggested a substantial ecological reassembly of the human gut microbiome in response to fasting.

### Identification of fasting-resistance and fasting-sensitive bacteria

**Figure 4:**
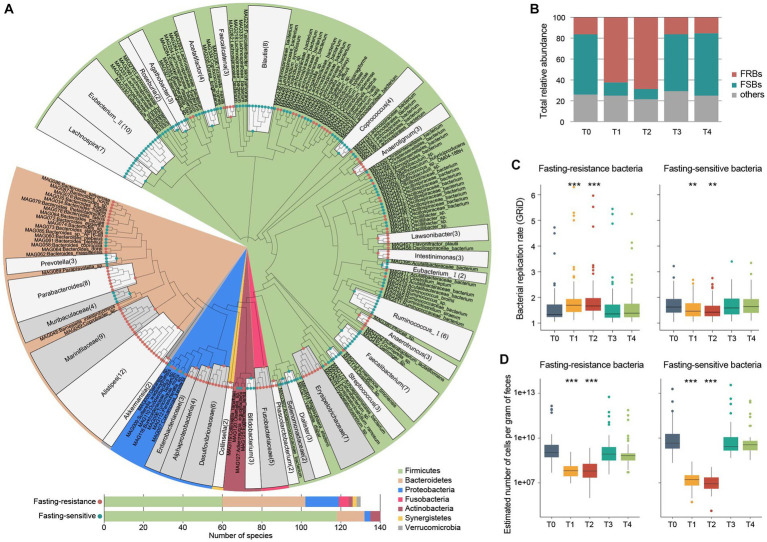
Overall representation of the fasting-resistance and fasting-sensitive bacteria. **(A)** Taxonomic characterization of 130 fasting-resistance bacteria (FRBs) and 140 fasting-sensitive bacteria (FSBs). Upper panel, phylogenetic tree showing the taxonomic assignments of the FRBs and FSBs. The adjacent species belonging to the same genus/family are grouped. Bottom panel, barplot showing the number of FRBs and FSBs for each bacterial phylum. **(B)** Total relative abundances of FRBs and FSBs in the gut microbiota at five timepoints. **(C)** Changes in the bacterial replication rate of FRBs (left panel) and FSBs (right panel) of the participants at five timepoints. Boxes represent the interquartile range between the first and third quartiles and the median (internal line). Whiskers denote the lowest and highest values within 1.5 times the range of the first and third quartiles, respectively; dots represent outlier samples beyond the whiskers. **(D)** Changes in the estimated number of cells per gram of feces in FRBs (left panel) and FSBs (right panel) among the participants at five timepoints. Significance levels compared with samples at T0 (Wilcoxon rank-sum test): *, *p* < 0.05; **, *p* < 0.01; ***, *p* < 0.001.

To identify actively or inactively microbes during fasting, we compared the relative abundance profiles of gut species between the fasting period and non-fasting timepoints. This analysis showed that 270 of 433 species significantly differed in relative abundances between the two stages (Figure 4A; Supplementary Figure S4; Supplementary Table S5), including 130 species that increase during fasting and 140 species decrease. These species were defined as fasting-resistant bacteria (FRBs) and fasting-sensitive bacteria (FSBs), respectively. On average for all individuals, the gross relative abundance of FRBs increased from16.4% at the baseline to 62.6 and 68.7% at T1 and T2, respectively (Figure 4B); considering that the microbial load of feces was substantially reduced during the fasting period, the total cell numbers of these FRBs still decreased to some extent (estimated number of cells per gram of feces, T0 vs. T_1/2_ = 1.1×10^9^ vs. 6.5×10^7^, *p* = 5.5×10^−12^; Figure 4D). In addition, we calculated the bacterial replication rates of each species to evaluate their growth status and found that the replication rate of FRBs is significantly increased at both T1 and T2 timepoints compared with the baseline (*p* < 0.001; Figure 4C). These results indicated that the FRBs can indeed be activated during fasting. Inversely, the gross relative abundance, estimated number of cells, and replication rate of FSRs were remarkedly reduced during the fasting period (Figures 4B–D).

Taxonomically, the FRBs included members belonged to Firmicutes (*n* = 60), Bacteroidetes (*n* = 42), Proteobacteria (*n* = 17), Fusobacteria (*n* = 5), Actinobacteria (*n* = 2), Synergistetes (*n* = 2), and Verrucomicrobia (*n* = 2); whereas the FSBs were mainly composed by Firmicutes members (*n* = 118), followed by members of Bacteroidetes (*n* = 14), Actinobacteria (*n* = 5), and Proteobacteria (*n* = 3; Figure 4A). At the family and genus levels, we found that the species belonged to *Alistipes*, Marinifilaceae, *Parabacteroides*, Erysipelotrichaceae, *Intestinimonas*, *Lawsonibacter*, and *Faecalicatena* were appeared in the FRBs, while the species belonged to *Prevotella*, Muribaculaceae, *Faecalibacterium*, *Ruminococcus_I*, *Coprococcus*, *Blautia*, *Eubacterium_II*, and *Lachnospira* were FSBs (Figure 4A; Supplementary Table S5). Bacteroidetes are primary degraders of polysaccharides in the human gut (Lapebie et al., 2019), and of these the *Prevotella* members are inclined to plant glycans such as fiber, xylan, starch, and pectin (David et al., 2014; Chen et al., 2017; Fehlner-Peach et al., 2019). Thus, the depletion of *Prevotella* members during fasting is consistent with the findings in the function aspect showing a substantial reduction of the degradation capacity of plant-derived polysaccharides. Firmicutes have the highest species diversity in the gut, and they are involved in a wide variety of important functions in the gut ecosystem, including metabolism/production of amino acids, vitamins, and SCFAs (Dai et al., 2011; Houtman et al., 2022). The higher proportion of FSBs in Firmicutes species during fasting suggested that they are vulnerable to environmental influences. In particular, some fasting-resistance Firmicutes such as Erysipelotrichaceae spp. are potential pathogens associated with human diseases (Kaakoush, 2015). On the other hand, certain Proteobacteria species such as Enterobacteriaceae and Desulfovibrionaceae members are more likely to survive or proliferate in the severe fasting environment (Figure 4A), which may be related to their strong environmental adaptability (David et al., 2014; Walterson and Stavrinides, 2015). For both Bacteroidetes and Firmicutes, we found that the higher bacterial genome size and number of functional orthologs are strongly beneficial to the fasting resistance (Supplementary Figure S5), suggesting that higher metabolic potential is also a strategy for survival during fasting.

### Functional characteristics and host dependence of FRBs and FSBs

**Figure 5:**
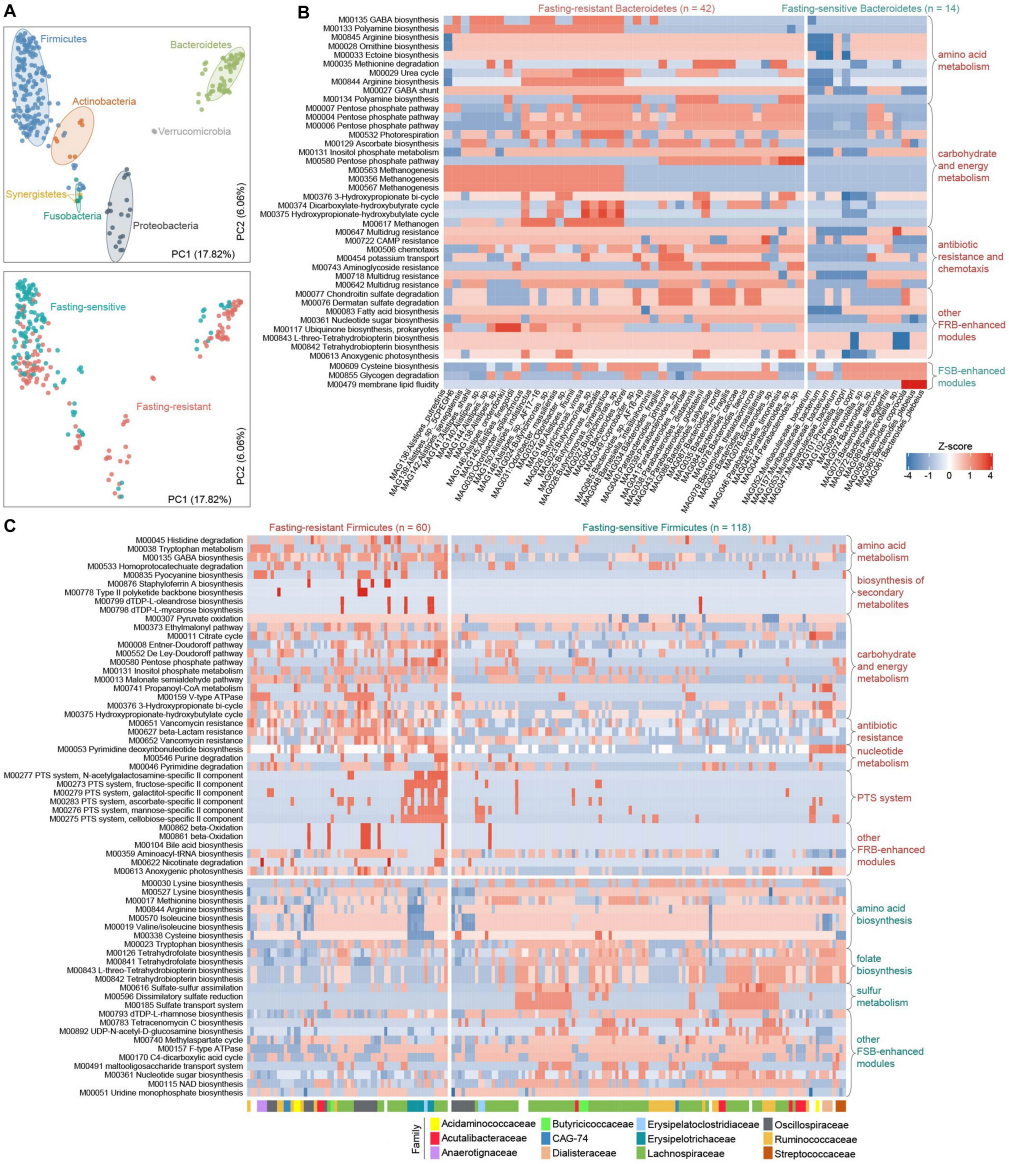
Functional configuration of the fasting-resistance and fasting-sensitive bacteria. **(A)** Principal coordinates analysis (PCoA) of the functional profiles of fasting-resistance bacteria (FRBs) and fasting-sensitive bacteria (FSBs). Bacterial species are shown at the first and second principal coordinates (PC1 and PC2), and the ratio of variance contributed by these two PCs is shown. Species are colored based on their phylogeny at the phylum level (upper panel) or the stratification of FSBs and FRBs (bottom panel). Ellipsoids represent a 95% confidence interval surrounding each phylum. **(B,C)** Heatmap showing the abundances of the differential KEGG modules between FRBs and FSBs belonging to Bacteroidetes **(B)** and Firmicutes **(C)**. Each row represents a KEGG module, and each column represents a species. For **(B)**, the family-level taxonomic assignments of each species are shown. In the heatmap, the displayed modules are those with a Wilcoxon rank-sum test *q*-value of less than 0.05.

To elaborate on how the fasting response of gut bacteria is linked to their genomic and functional scopes, we first compared the KEGG and CAZy profiles between FSBs and FRBs using multivariate analyzes. In agreement with previous studies (Forster et al., 2019), the functional composition of gut bacteria was primarily determined by their phylogenetic relationship at the phylum level (effect sizes 36 and 30% for the KEGG and CAZy profiles, respectively, Permutational multivariate analysis of variance (PERMANOVA) *p* < 0.001; Figure 5A; Supplementary Figure S6). Stratification of FSBs and FRBs explained only 7% (PERMANOVA *p* < 0.001) variation in the functional composition. This finding raised us to compare the functional characteristics of FRBs and FSBs within each phylum to reduce the impact of the phylogeny.

#### Bacteroidetes

We identified 39 modules that significantly enhanced in module abundance (NumberofKEGGorthologs×modulecompletionratio; see Methods) in the fasting-resistance Bacteroidetes, whereas only 3 modules were enhanced in the FSBs (*q* < 0.05; Figure 5B). The FRB-enhanced modules had more widely participated in amino acid metabolism (e.g., biosynthesis of arginine, polyamine, and ornithine and degradation of methionine), carbohydrate metabolism (mainly involved in PPP), energy metabolism (e.g., methanogenesis), antibiotic resistance and chemotaxis, and some modules involved environmental information processing and metabolism of other substances (e.g., chondroitin, dermatan, tetrahydrobiopterin, and ubiquinone). The higher frequency of PPP modules in FRBs, especially in *Parabacteroides* and *Bacteroides* members, was in agreement with the observations in overall gut microbiota function. And particularly, the PPP product, NAPDH, is a source of multiple sugar molecules that are required for the biosynthesis of nucleic acids and amino acids (Xue et al., 2017; Xu et al., 2018; Zhan et al., 2019), probably linking to the high biosynthesis capacity of arginine, ornithine, ectoine, and nucleotide sugar in fasting-resistance *Bacteroidetes* species. The methanogenesis modules, which may help to promote the carbon cycle in the gut microbiota under malnutrition (Million et al., 2016), were mainly encoded by members of fasting-resistance *Butyricimonas*, *Alistipes*, and *Parabacteroides* spp. Another striking phenomenon is the enhancement of two glycosaminoglycan (GAG) degradation modules (i.e., chondroitin and dermatan sulfate degradation) in FRBs. These modules were mainly encoded by members of *Bacteroides* and *Parabacteroides*. GAG, a type of mucopolysaccharides, is the major component of the extracellular matrix in animals that showed various physiological functions in the human gut (Cartmell et al., 2017; Kawai et al., 2018). This result suggested that, similar to carbohydrates, some bacteria can survive by using proteins from the hosts during the fasting period. Consistent with GAGs, the host glycoprotein mucin was also degraded by fasting-resistance *Parabacteroides* and *Bacteroides*.

#### Firmicutes

We identified 80 and 25 modules that significantly enhanced in module abundance in the FRBs and FSBs (*q* < 0.05; Figure 5C), respectively. Consistent with the *Bacteroidetes*, the FRB *Firmicutes* also more widely participated in carbohydrate and energy metabolism, especially the central metabolism modules involving to citrate cycle, PPP, Entner-Doudoroff pathway, and De Ley-Doudoroff pathway. Meanwhile, a large proportion (47/80, 58.8%) of FRB-enhanced modules were involved in environmental information processing such as transport system, two-component system, phosphotransferase system (PTS), and multidrug resistance (Supplementary Figure S7). Particularly, some transporters for uptake of plant carbohydrates such as fructose (M00273), ascorbate (M00283), mannose (M00276), and cellobiose (M00275) in gut microbiota were more abundant to be encoded in FRBs (Figure 5C). Compared with the FSBs, the higher capacity of carbohydrate/energy metabolism and environmental information processing in FRBs may involve in greater tolerance to severe oligotrophic environments during fasting. The other FRB-enhanced modules had participated in amino acid metabolism (e.g., degradation of histidine and tryptophan), biosynthesis of secondary metabolites, and nucleotide metabolism, while the FSB-enhanced modules were more widely distributed in sulfur metabolism and biosynthesis of amino acids (e.g., lysine, isoleucine, and methionine), folate, and other substances (e.g., uridine, nucleotide sugar, NAD) (Figure 5C). A notable function that is enriched in the FSBs is folate biosynthesis, for which we found that the major folate producers such as Lachnospiraceae members and *Streptococcus* spp. were sensitive to fasting. These findings were different from the observations in humans with the carbohydrate-restricted diet (Mardinoglu et al., 2018), probably due to differences in intervention patterns between the carbohydrate-restricted diet and our water-only fasting.

### Fasting-responded species contribute to serum and urine metabolome changes

**Figure 6:**
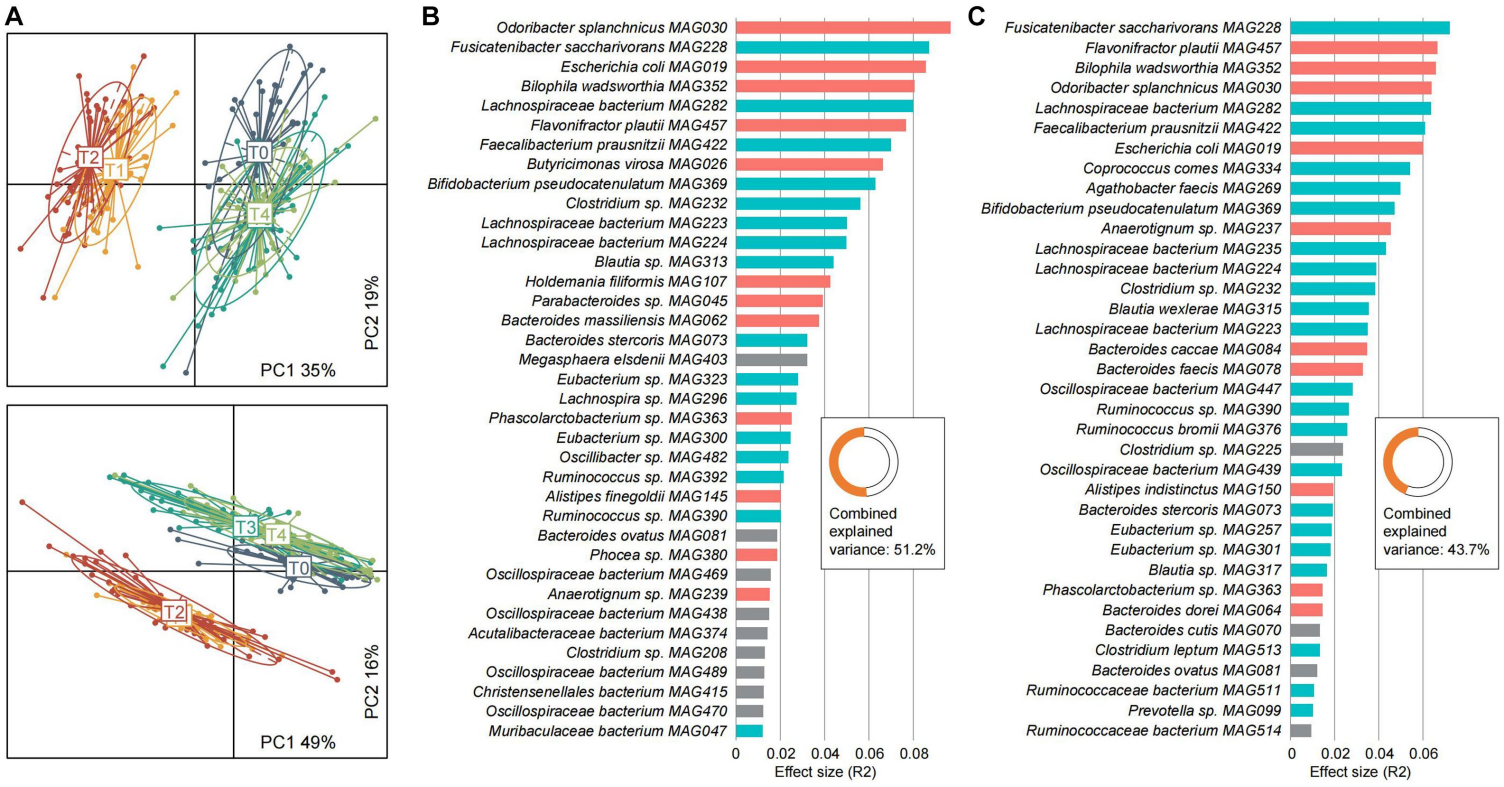
Characterization of the serum and urine metabolomes and their associations with gut microbiota. **(A)** Principal coordinates analysis (PCoA) of the serum metabolome (upper panel) and urine metabolome (bottom panel) of all samples. Samples are shown at the first and second principal coordinates (PC1 and PC2), and the ratio of variance contributed by these two PCs is shown. Lines connect samples that belonged to the same timepoint, and ellipsoids represent a 95% confidence interval surrounding each timepoint. **(B,C)** Barplots showing the effect sizes of gut species on the serum **(B)** and urine **(C)** metabolomes. The combined effect sizes of all species are shown in the inset panel. Colors represent the fasting-resistance (red), fasting-sensitive (blue), and other bacteria (gray).

To explore the dynamics of the host metabolome across the fasting process, we performed untargeted mass spectrometry (MS) analysis of the serum and urine samples of 46 participants at five timepoints. The serum and urine metabolomes were profiled based on 1,424 and 1,820 annotated metabolites, respectively, from the MS datasets. PCoA analysis revealed that both the serum and urine metabolomes are substantially altered during the fasting period and could largely recover at the T3 and T4 timepoints (Figure 6A). During the fasting period, 52.0% of serum metabolites and 66.8% of urine metabolites had significant abundances compared with baseline (Supplementary Tables S6, S7). During fasting, benzenoids, lipids and lipid-like molecules, organic acids and derivatives, and organoheterocyclic compounds were enhanced in individuals’ serum, while many of these metabolites were reduced in urine (Supplementary Figure S8).

Furthermore, we carried out an inter-omics analysis to quantify the strength of association between the gut microbiome and serum and urine metabolomes. Univariate analysis identified 37 gut species that independently impact the serum metabolome, as well as 36 gut species that impact the urine metabolome (Figures 6B,C). Several fasting-associated species, such as the fasting-resistance *Odoribacter splanchnicus MAG030*, *Escherichia coli MAG019*, *Bilophila wadsworthia MAG352*, and *Flavonifractor plautii MAG457* and the fasting-sensitive *Fusicatenibacter saccharivorans MAG228*, *Lachnospiraceae bacterium MAG282*, and *Faecalibacterium prausnitzii MAG422* had significant effect sizes on both serum and urine metabolomes, suggesting their importance in shaping the host metabolism homeostasis. Moreover, multivariate analysis showed that the effect of the gut microbiome on metabolic profiles was considerable, as it accounted for 51.2 and 43.7% of the serum and urine metabolome variances, respectively. These findings suggested that the gut microbiota as well as the fasting-responded species appear to be an important determinant of the host metabolic landscape.

Finally, we also assess the association between gut microbial populations and clinical characteristics. Correlation analysis identified 2,195 significant pairwise correlations (Spearman’s |ρ| > 0.35, *q* < 0.05), involving 224 gut species and 45 clinical characteristics (Supplementary Figure S9). Several fasting-resistance species, such as *Desulfovibrio desulfuricans MAG347*, *Flavonifractor plautii MAG457*, and *Intestinimonas massiliensis MAG458*, exhibited a significant positive correlation with serum immunoglobulin levels, suggesting their importance in the host’s immune response during fasting. In addition, the fasting-resistance species fasting-resistance species were frequently positively correlated with indicators like bilirubin, lipoproteins, and creatinine, while the fasting-sensitive species more commonly showed significant positive correlations with indicators such as prealbumin, ApoB100/ApoAI ratio, and fasting blood glucose. These findings suggested that the fasting-responded species may play important roles in influencing various aspects of the host’s health.

## Conclusion

In summary, the present study investigated the patterns of gut microbiota alterations in a cohort of 46 obese volunteers across a 7-days prolonged fasting and recovery phase. The gut microbial diversity, composition, and estimated bacterial cell number revealed substantial changes during the fasting, with a rapid return to almost original levels after fasting. In terms of microbial function, the ecological pattern of the gut microbiota had also undergone rapid adaptive changes. The capacity of carbohydrate degradation, especially almost all plant-derived polysaccharides (e.g., sucrose, starch, rhamnose, and pectin), were reduced in the gut microbiota during fasting, while other metabolic abilities such as the degradation of glycoproteins, amino acids, lipids, and organic acid metabolism, were enhanced. Moreover, we found that changes in the gut microbiota were also, to a large extent, reflected in the serum and urine metabolites, highlighting the important role of the gut microbiota in this procedure. We identified 270 fasting-responded gut species, including 130 FRBs that still have activity (e.g., increase in bacterial replication rate) during fasting and 140 FSBs have been suppressed by fasting. FRBs included many Bacteroidetes members that have been extensively involved in the metabolism of various nutrients, as well as some members of Proteobacteria and Fusobacteria, while the FSBs were mostly Firmicutes members. Functional comparison between FRBs and FSBs untangled the associations of taxon-specific functions (e.g., pentose phosphate pathway modules, antibiotic resistance and chemotaxis, glycosaminoglycan degradation, and folate biosynthesis) with fasting. Together, our findings explained the main responses and strategies for gut bacterium to respond to the extreme nutritional deficiency condition, the results from this study provided guidance for subsequent bacterial ecology (e.g., bacterial cultivation) and clinical intervention (e.g., very low-calorie intake therapy) researches and will enhance our understanding and interpretation of the microbiota-human inter-associations.

## Methods

### Ethics approval and consent to participate

This study was approved by the Medical Ethics Committee of Beijing University of Chinese Medicine (2017BZHYLL0404). A written informed consent was obtained from each individual. To avoid the potential risk on the participants, we reference the safety and quality standards of an expert panel update of the 2002 consensus guidelines of fasting therapy (Wilhelmi de Toledo et al., 2013).

### Study design and sampling

This study was designed as a controlled trial by comparing the differences in participants’ physical status before and after the study. Volunteers were recruited between November and December 2016. This study included a 7-days medically supervised water-only fasting procedure, the subsequent recovery phase and follow-up phase (Supplementary Figure S1B). There were five timepoints: T0 (pre-fasting, Day 0), T1 (the 3rd day of fasting, Day 3), T2 (the 7th day of fasting, Day 7), T3 (the 7th day after fasting, Day 14), T4 (the 14th day after fasting, Day 21). The dinner of D0 was supplied only an apple (≤300 kcal). In the fasting period (Day 1 to Day 7), all participants received standard medical care as determined by their individual requirements. Thereafter, participants were allowed unrestricted amounts of natural drinking water (0 kcal, NONGFU SPRING, Guangdong, China), and were advised to drink at least 2.5 L daily. Traditional Chinese meditation and exercises were performed systematically every day. In the recovery period (Day 8-Day 14), participants had a low-calorie diet during the first 3 days, followed by gradually recover to normal diet in the next 4 days. The dietary calories consumed on Day 8, Day 9, and Day 10 were approximately 800 kcal, 1,000 kcal, and 1,200 kcal, respectively. From Day 11 to Day 14, the caloric intake gradually increased to 1,600 kcal. The source of calories was millet, with a caloric value of 360 kcal per 100 g, obtained from the China National Cereals, Oils and Foodstuffs Corporation in Beijing, China. During the follow-up period from Day 15 to Day 21, no intervention was conducted except for a questionnaire survey and telephone return visit.

The inclusion criteria in this trial were: aged from 20 to 70, central obesity (waist circumference: male ≥90 cm, female ≥80 cm), body mass index (BMI) ≥ 25 kg/m^2^, or concurrently diagnosed with one of the following three items: (1) elevated triglyceride (TG) level > 1.7 mmoL/L, or treated correspondingly; (2) decreased high-density lipoprotein-cholesterol (HDL-C) level (male <0.9 mmoL/L, female <1.1 mmoL/L), or treated correspondingly; (3) elevated fasting plasma glucose (FPG) (≥ 5.6 mmoL/L), or diagnosed with type 2 diabetes or received appropriate treatment. All participants were volunteered to join fasting and the trial. Exclusion criteria included: patients with type 1 diabetes mellitus, pregnant or lactating women, patients with secondary obesity or severe heart, liver and kidney dysfunction and other serious illness as well as patients undergoing any weight reduction treatment in recent 3 months.

All participants were weighed in light clothing without shoes at the same time in the morning at two time points T0 and T2. Body weight, visceral fat rate and body fate rate were measured by full body sensor body composition monitor and scale (Omron HBF-701). BMI was calculated as weight [kg]/height^2^ [m^2^]. Waist circumference (WC) was measured at the midpoint between the lower border of the rib cage and the top of the lateral border of the iliac crest by study physicians. Blood pressure was measured in the right arm using the sphygmomanometer (Yuwell, type A) after participants had rested in a seated position for 5 min. Blood and urine samples were collected in the morning after an overnight fast (T0, T3, T4) or during the fasting (T1, T2). Blood samples for clinical were analyzed in 2 h at the hospital. Serum samples were obtained by centrifugation at 3000 rpm for 10 min and immediately stored at −80°C until analysis. Urine samples were stored at −80°C without any preservatives. Feces samples were collected from all participants in the morning at five timepoints (T0-T4) were immediately placed into dry ice containers for transportation to the laboratory and preserved at −80°C until further processing. Adverse events were monitored by standardized questionnaires at T0 and T2 points.

### Clinical laboratory tests

Complete blood count was measured using a hematology automated analyzer (Sysmex XE-2100, Sysmex XT-4000i). Serum alanine transaminase (ALT), aspartate transaminase (AST), blood urea nitrogen (BUN), creatinine (CRE), glucose, triglycerides (TG), total cholesterol (TC), high-density lipoprotein cholesterol (HDL-C), and low-density lipoprotein cholesterol (LDL-C), immunoglobulin G (IgG), immunoglobulin A (IgA), immunoglobulin M (IgM), complement component 3 (C3) and complement component 4 (C4) were measured using an autoanalyzer (Hitachi 7,600–110, Beckman AU-680).

### Qualitative study

Semi-structured interviews or focus group interviews were conducted among 14 individuals in our study by Qing Dao television station from D8 to D15, and there were 6 individuals wrote down their experiences during fasting and shared to us at D14. These verbatim transcripts were made and analyzed by two and more coders via NVivo software (version 11) using content analysis method.

### Serum and urine metabolomics

LC-HRMS was performed on a Waters UPLC I-class system equipped with a binary solvent delivery manager and a sample manager, coupled with a Waters VION IMS Q-TOF Mass Spectrometer equipped with an electrospray interface (Waters Corporation, Milford, United States).

LC Conditions: Column: Acquity BEH C18 column (100 mm × 2.1 mm i.d., 1.7 μm, Waters, Milford, United States). Solvent: The column was maintained at 50°C and separation was achieved using the following gradient: 1%B–1% B over 0–1.0 min,1%B–20% B over 1.0–5.5 min, 20%B–30%B over 5.5–6.0 min; 30%B–35%B over 6.0–8.5 min; 35%B–70%B over 8.5–10.5 min; 70%B–100%B over 10.5–11.0 min; 100%B–100%B over 11.0–13.0 min;100%B–1%B over13.0–13.1 min; and 13.1–15.0 min holding at 1% B at a flow rate of 0.40 mL/min, where B is acetonitrile (0.1% (v/v) formic acid) and A is aqueous formic acid (0.1% (v/v) formic acid). Injection Volume was 3.00 μL and Column Temperature was set at 50.0°C.

The mass spectrometric data was collected using a Waters VION IMS Q-TOF Mass Spectrometer equipped with an electrospray ionization (ESI) source operating in either positive or negative ion mode. The source temperature and desolvation temperature was set at 120°C and 500°C, respectively, with a desolvation gas flow of 900 L/h. Centroid data was collected from 50 to 1,000 m/z with a scan time of 0.1 s and interscan delay of 0.02 s over a 13 min analysis time.

QC sample was prepared by mixing aliquots of the all samples to be a pooled sample, and then analyzed using the same method with the analytic samples. The QCs were injected at regular intervals (every ten samples) throughout the analytical run to provide a set of data from which repeatability can be assessed.

The UPLC–Q-TOF/MS raw data were analyzed by progenesis QI (Waters Corporation, Milford, United States) software using the following parameters. The parameters used were retention time(RT) range 0.5–14.0 min, mass range 50–1,000 Da, mass tolerance 0.01 Da. Isotopic peaks were excluded for analysis, noise elimination level was set at 10.00, minimum intensity was set to 15% of base peak intensity and, finally, RT tolerance was set at 0.01 min. The Excel file was obtained with three-dimensional data sets including m/z, peak RT and peak intensities, and RT–m/z pairs were used as the identifier for each ion. The resulting matrix was further reduced by removing any peaks with missing value (ion intensity = 0) in more than 60% samples. The internal standard was used for data QC (reproducibility). The positive and negative data were combined to get a combined data set which was imported into SIMCA-P+ 14.0 software package (Umetrics, Umeå, Sweden).

### DNA extraction, library construction, and whole-metagenome shotgun sequencing

DNA for metagenomics sequencing was extracted from fecal samples by using the E.Z.N.A.® fecal DNA Kit (Omega Bio-tek, Norcross, GA, United States) according to the manufacturer’s protocols. The DNA concentration and purity were quantified with TBS-380 and NanoDrop2000, respectively. DNA quality was examined with the 1% agarose gel electrophoresis system.

DNA was fragmented to an average size of about 300 bp using Covaris M220 (Gene Company Limited, China) for paired-end library construction. Paired-end library was prepared by using the TruSeq™ DNA Sample Prep Kit (Illumina, San Diego, CA, United States). Adapters containing the full complement of sequencing primer hybridization sites were ligated to the Blunt-end fragments. Paired-end sequencing was performed on the Illumina HiSeq4000 platform (Illumina Inc., San Diego, CA, United States) at Majorbio Bio-Pharm Technology Co., Ltd. (Shanghai, China).

### Sequencing data quality control

Initial base calling of metagenomic sequencing data was performed using Illumina HiSeq4000 platform with system default parameters. For each metagenomic sample, low-quality paired-end reads were discarded in pair using fastp (Chen et al., 2018) based on any of the following criteria: (1) read with more than 30% bases that be estimated with error rate > 1%; (2) read with ambiguous “N” > 5 bp; (3) read less than 90 bp; (4) read less than 30% low complexity. Furthermore, human genomic DNA reads were identified via Bowtie2 (Langmead and Salzberg, 2012), and were removed if it shared >95% sequence with the human genome reference sequence (GRCh38). The remaining high-quality paired-end reads from metagenomic samples were used for further analysis.

### Metagenomic-assembled genomes

For metagenomic samples, we carried out single-sample assembly using MEGAHIT (Li et al., 2015) (option “--k-list 21,41,61,81,101,121,141”), and then assembled contigs longer than 2 kbp in each sample were binned by MetaBAT2 (Kang et al., 2019) with default parameters, leading to 12,291 raw bins of over 200 kbp. In addition, to improve assembly quality, multiple metagenomic samples from the same individual were merged into a new metagenomic sample, and intra-individual assembly and binning were performed based on these merged samples, resulting in 6,800 raw bins of over 200 kbp. The completeness and contamination of all 19,091 raw bins were assessed by CheckM (Parks et al., 2015). For low-quality raw bins with <50% completeness and < 5% contamination, an additional approach was used to improve the quality of these raw bins. Firstly, the taxonomic classification of low-quality raw bins was carried out using the GTDB-Tk toolkit (Chaumeil et al., 2020). Meanwhile, the sequencing depth for each raw bin was defined by mapping the high-quality reads from the corresponding metagenomic sample back to the bin with Bowtie2 (Langmead and Salzberg, 2012) and SAMtools (Li et al., 2009). Lastly, raw bins were merged if they met all of the following criteria: (1) they were recovered from the same metagenomic sample; (2) they had a similar sequencing depth (±10%) and GC content (±2%); (3) they had an identical taxonomic assignment at the species level. The completeness and contamination of merged bins were re-assessed by CheckM (Parks et al., 2015). Finally, we obtained a collection of 5,263 high-quality MAGs (≥90% complete and ≤ 5% contamination). All high-quality MAGs were clustered using dRep (Olm et al., 2017), leading to a de-replicated database of 433 species-level MAG. The taxonomic classification of these species-level MAGs was carried out using GTDB-Tk (Chaumeil et al., 2020). Phylogenetic analysis was performed using PhyloPhlAn (Asnicar et al., 2020), and visualized by iTOL^1^ (Letunic and Bork, 2021).

To generate microbial community profiles, the high-quality paired-end reads of each sample were firstly aligned against the 433 species-level MAGs using Bowtie2 (Langmead and Salzberg, 2012). For the species-level profile, the relative abundance of each MAG was the number of mapped reads for each MAG divided by genomic length and the total number of mapped reads for each sample. For taxonomic profiles of the higher rank levels, the abundance of each taxon was calculated by summing up the relative abundance of MAGs from the same taxon. In addition, we calculated bacterial replication rates of MAGs in each sample to analyze the dynamics of bacterial growth via GRiD (Emiola and Oh, 2018).

### Functional analysis

Genes of 433 species-level MAGs were predicted by Prodigal (Hyatt et al., 2010) with option ‘-p meta’. Functional annotation of the protein-coding genes was performed by assigning the protein sequences to the KEGG (Kyoto Encyclopedia of Genes and Genomes) database (Kanehisa et al., 2016) and using DIAMOND (Buchfink et al., 2015) with options ‘--evalue 1e-10 --query-cover 50’. Each protein was assigned a KEGG orthologue (KO) with the highest score. Annotation of carbohydrate-active enzymes (CAZymes) was performed by aligning the protein sequence of each MAGs against the CAZy database^2^ using DIAMOND (*e*-value <1e-10 and covering >50% of the protein length).

To generate functional profiles of each sample, all putative protein-coding genes were firstly dereplicated by CD-HIT based on nucleotide level similarity >95% and sequence overlap >90%. Then, the high-quality paired-end reads of each sample were aligned against these dereplicated protein-coding genes using Bowtie2. The relative abundance of each gene was the number of mapped reads for each gene divided by gene size and the total number of mapped reads for each sample. For functional profiles, the abundance of each KO (or CAZy family) was calculated as the sum of abundance of genes from the same functional ortholog divided by abundance of all genes with KO (or CAZy family) assignment. In addition, to explore higher-order functions, KOs were assigned into gut metabolic modules (GMMs) (Vieira-Silva et al., 2016), a manually curated metabolic module framework. For GMM profiles of each sample, each GMM abundance was calculated as the median abundance of KOs assigned to the GMM, and then assigned the value of 0 if the number of KOs assigned to this GMM was less than half of total number of KOs in this GMM.

To generate functional profiles of each MAG, each KO (or CAZy family) abundance was calculated as the number of gene from the same functional ortholog in this MAG. KOs were assigned into KEGG modules based on the KEGG website^3^ (Kanehisa et al., 2016). To explore higher-order functions of genomes, we calculated the module completion ratio (MCR) of each MAG based on a Boolean algebra-like equation (Takami et al., 2012) using custom scripts.

### Statistical analysis

Statistical analyzes were performed on the R platform. The differences of clinical data at five time points were compared based on Student’s *t*-test. For the alpha diversities, the Shannon diversity index was calculated via the function ‘diversity’, and the observed number of species was calculated as the count of unique species-level MAGs in each sample. The Bray-Curtis distance between samples was evaluated based on species-level profiles by the function ‘vegdist’. (PCoA) based on Bray-Curtis distance was performed using the function ‘pcoa’, and normal confidence ellipses were computed using the function ‘stat_ellipse’ with parameters: geom of polygon and level of 0.8. PERMANOVA analysis was performed using the function ‘adonis’, and *adonis* R (Mardinoglu et al., 2018) was adjusted using the function ‘RsquareAdj’. Statistical significance between two groups was assessed using the Wilcoxon rank-sum test via the function ‘wilcox.test’. For comparisons involving multiple groups, the Kruskal-Wallis test was employed, and calculations were conducted using the ‘kruskal.test’ function. The *p*-values obtained were adjusted into q values using the Benjamini-Hochberg method via the function ‘p.adjust’. To evaluate the impact of each bacterium on the metabolome, the ‘adonis’ function was used to estimate the “one-to-all” effect size (*R*^2^) between the relative abundance of each MAG and the overall metabolic profiles. For correlation analysis between gut microbiota and clinical characteristics, we calculated the Spearman Rank Correlation Coefficient using the ‘cor.test’ function with the ‘method = spearman’ option, and then adjusted the p-values to q-values using the Benjamini-Hochberg method via the ‘p.adjust’ function.

Data visualization was implemented using the function ‘ggplot2’.

### qPCR detection for bacteria counts from original feces

To count the total bacterial load in feces, we performed relative quantification analysis by quantitative PCR (ABI StepOne, United States) (Alavi et al., 2020). The standard curves were prepared using two internal reference strains (Use *Lactococcus lactis* NZ9000 to represent Gram-positive bacteria and *Escherichia coli* BL21 to represent Gram-negative bacteria). The bacterial genome DNA extracted from 5 mL of liquid medium was used as a reference sample, and total DNA extracted from 170 mg corresponding host feces was used as a test sample. The number of internal reference strains was determined using CFU counting by solid plates before bacterial genome extraction. Consistent with the reference (Alavi et al., 2020), universal primers used in the article listed below (forward: CTCCTACGGGAGGCAGCAG; reverse: TTACCGCGGCTGCTGGCAC). PCR enzymes and chemicals were used according to the manufacturer’s instructions (Innovgene, China). Each reaction was performed in triplicate. A 25 μL reaction system contained 1 μL of template DNA or sterile double distilled water (negative control), 12.5 μL of SYBR Premix (Innovgene, China), 0.5 μL of forward primer, 0.5 μL of reverse primer, and 10.5 μL of water. Amplifications were performed with the following reaction procedures: 1 cycle of predenaturation at 95°C for 3 min, followed by 39 cycles of denaturation at 95°C for 10 s and annealing at 55°C for 30 s. The total bacterial amount in test feces was calculated using the 2^−△△C(T)^ method (Alavi et al., 2020). For each fecal sample, we also estimated the bacterial amount of the fasting-resistance and fasting-sensitive bacteria, respectively. This was calculated as the sum of the relative abundances of all bacteria within each group divided by the total bacterial amount quantified for that sample using qPCR.

## Data availability statement

The data presented in the study are deposited in the National Center for Biotechnology Information Sequence Read Achieve (NCBI-SRA) repository, accession number PRJNA433058.

## Ethics statement

The studies involving humans were approved by Medical Ethics Committee of Beijing University of Chinese Medicine. The studies were conducted in accordance with the local legislation and institutional requirements. The participants provided their written informed consent to participate in this study.

## Author contributions

XS: Conceptualization, Writing – original draft. SL: Data curation, Formal analysis, Methodology, Writing – original draft. RG: Data curation, Formal analysis, Methodology, Writing – original draft. QY: Data curation, Writing – original draft. CL: Conceptualization, Writing – original draft. YZ: Data curation, Methodology, Writing – original draft. QL: Methodology, Writing – original draft. LW: Data curation, Writing – original draft. JM: Data curation, Methodology, Writing – original draft. WY: Data curation, Writing – original draft. LF: Data curation, Investigation, Writing – original draft. WS: Conceptualization, Writing – original draft.

## Footnotes

^1^ https://itol.embl.de/

^2^ http://www.cazy.org/

^3^ https://www.kegg.jp
