# Supplementary material for: Dynamics and ecological reassembly of the human gut microbiome and the host metabolome in response to prolonged fasting
Source: Front Microbiol. 2023 Oct 3;14:1265425. doi: 10.3389/fmicb.2023.1265425 (PMC10579591; doi:10.3389/fmicb.2023.1265425)
Supplement: Supplementary file 2 [file Data_Sheet_1.docx]

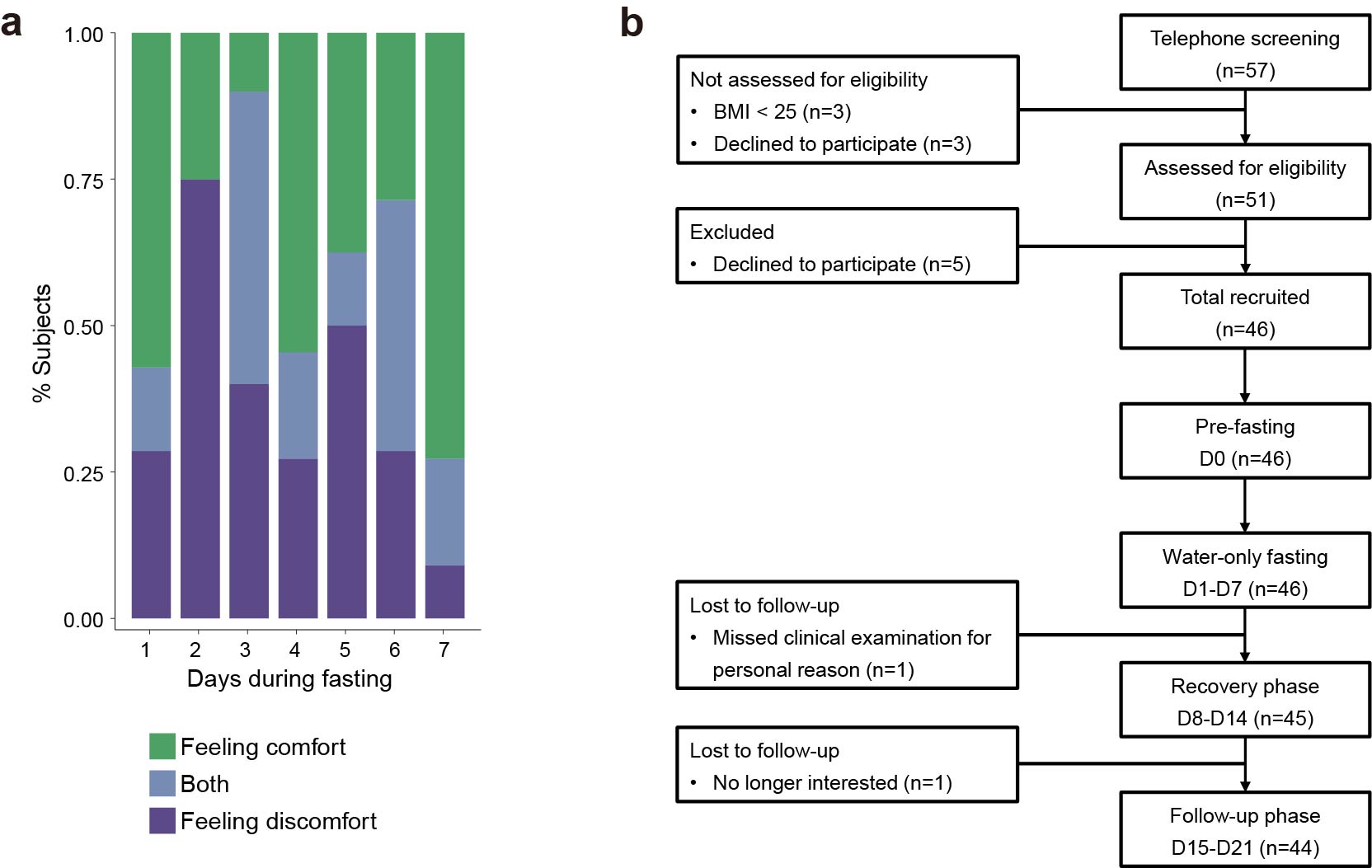


**Supplementary Figure 1| Study design, participants, and psychological evaluation.** **a,** Proportion of participants experiencing specific psychological responses to *bigu*. Interview and video materials were transformed into verbatim transcripts. Patient responses were sorted into two categories: comfort and discomfort. The comfort node included feeling energetic and happy; the discomfort node included food cravings, hunger, weakness, insomnia, stomach discomfort, negative moods, and related symptoms. The results showed that feelings of comfort increased with the duration of fasting. **b,** Flow diagram of the study.


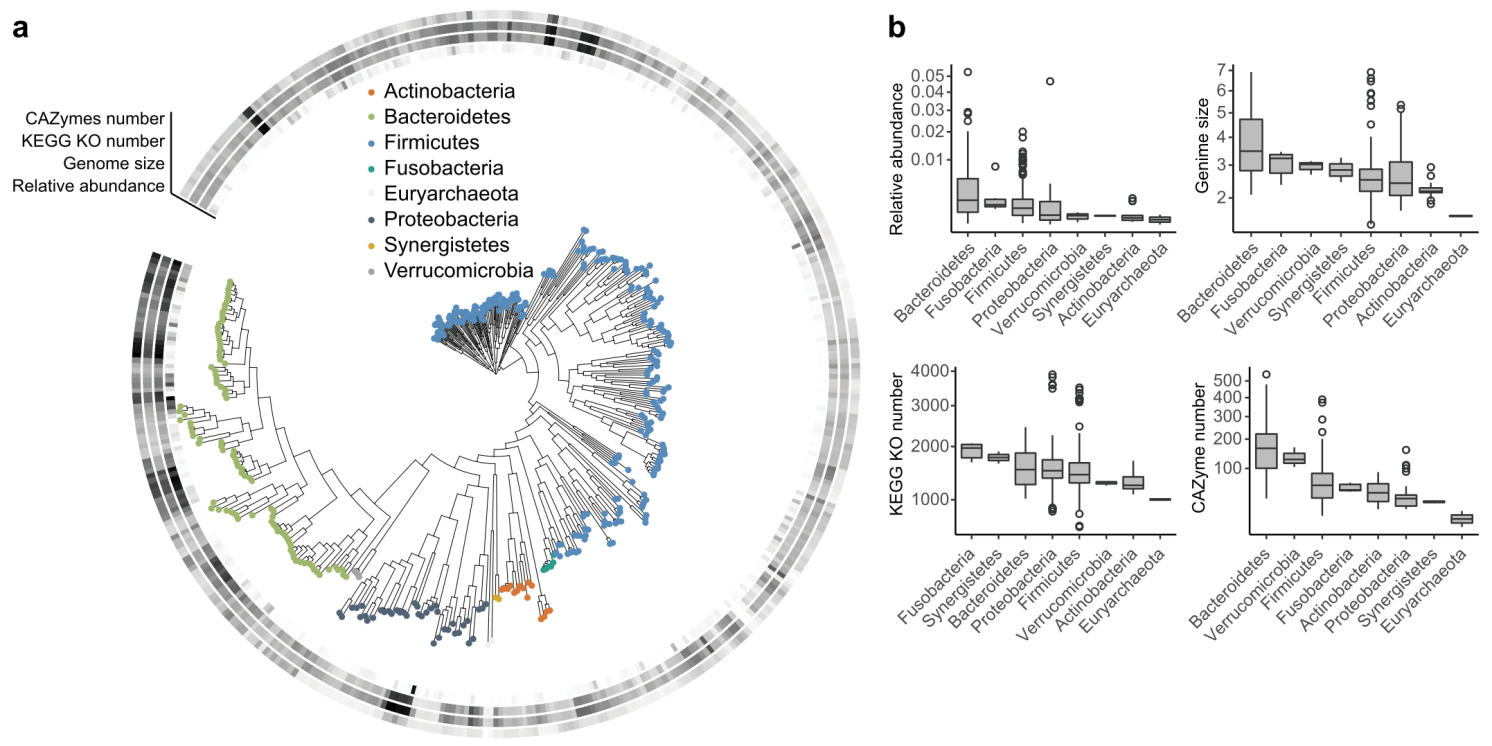


**Supplementary Figure 2| Characterization of 433 non-redundant gut microbial species reconstructed from the fecal metagenomes of this study. a,** Genome-wide phylogeny of 433 gut microbial species. Innermost circle shows the phylogenetic tree of 433 species. Colors of the phylogenetic tree represent the phylum level assignment of each species. The outer four circles (from inside to outside) represent the average relative abundance (among all investigated samples), genome size, number of KEGG orthologs and CAZymes of each species. **b,** Boxplots showing the average relative abundance (among all investigated samples), genome size, number of KEGG orthologs and CAZymes of all species, grouped by their phylum level phylogeny. Boxes represent the interquartile range between the first and third quartiles and the median (internal line). Whiskers denote the lowest and highest values within 1.5 times the range of the first and third quartiles, respectively; dots represent outlier samples beyond the whiskers. Detailed information of 433 species is shown in Supplementary Table 4.


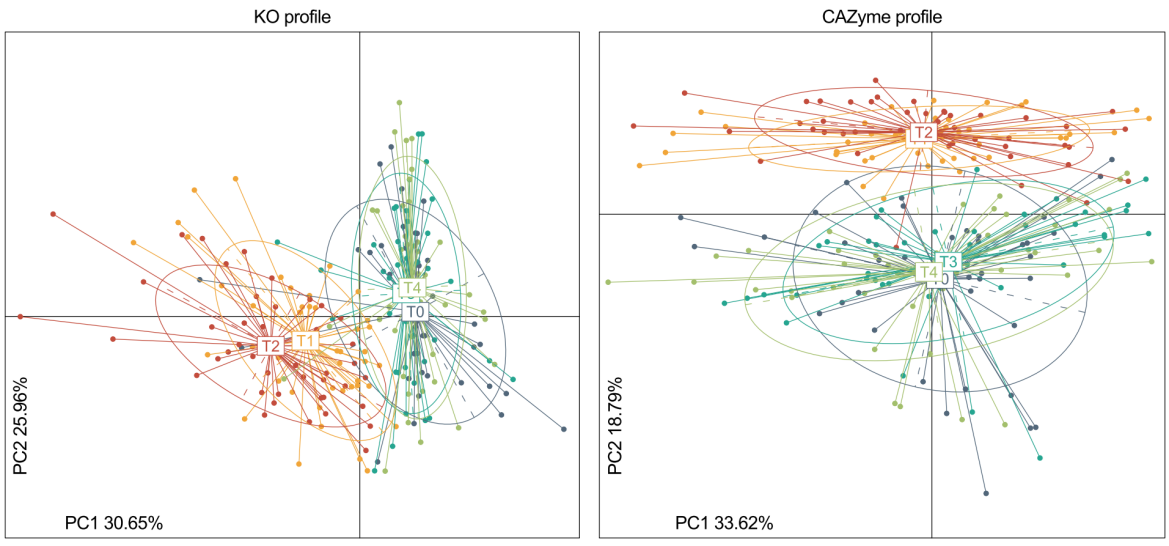


**Supplementary Figure 3| Principal coordinates analysis (PCoA) of the KEGG and CAZyme profiles of all samples.** Samples are shown at the first and second principal coordinates (PC1 and PC2), and the ratio of variance contributed by these two PCs is shown. Lines connect samples belonged to the same timepoint, and ellipsoids represent a 95% confidence interval surrounding each timepoint. b


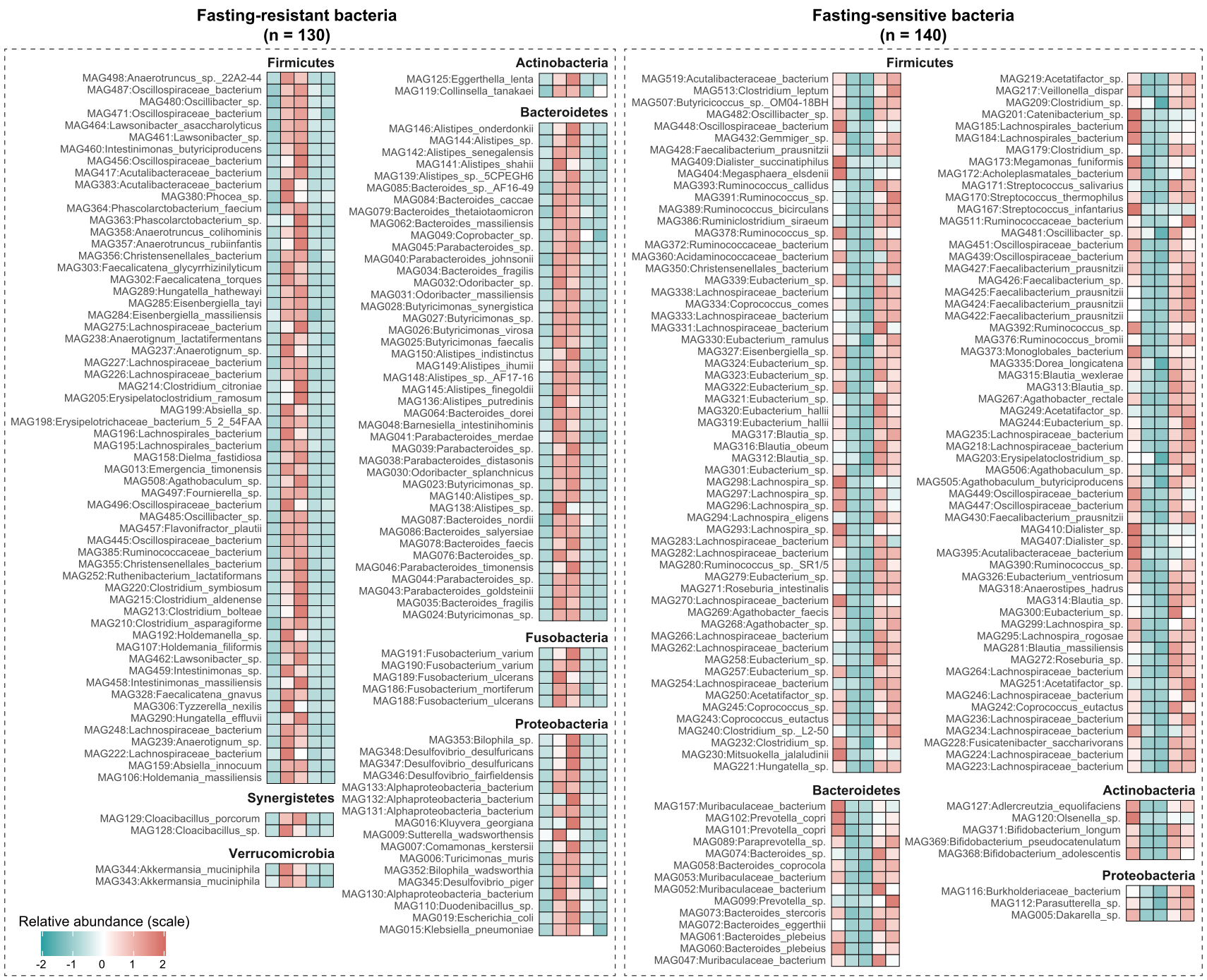


**Supplementary Figure 4| Heatmap showing the average relative abundances of fasting-resistance and fasting-sensitive bacteria at five timepoints.** Species are grouped based on their phylum level phylogeny.


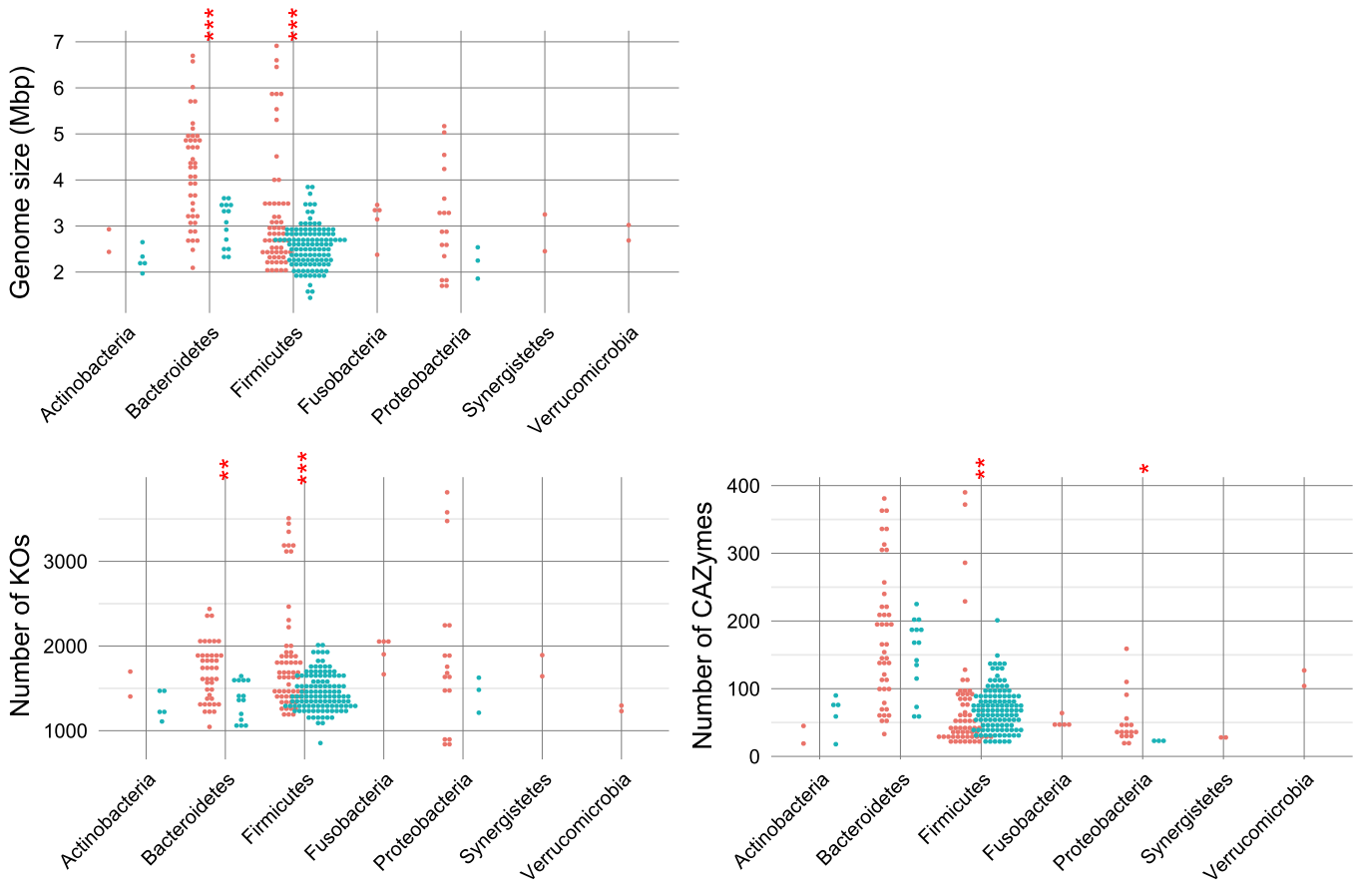


**Supplementary Figure 5| Distribution of genome sizes of fasting-resistance and fasting-sensitive bacteria.** Red and blue colors represent the fasting-resistance bacteria (FRBs) and fasting-sensitive bacteria (FSBs), respectively. Significance levels between FRBs and FSBs (Wilcoxon rank-sum test): *, *p*<0.05; **, *p*<0.01; ***, *p*<0.001.


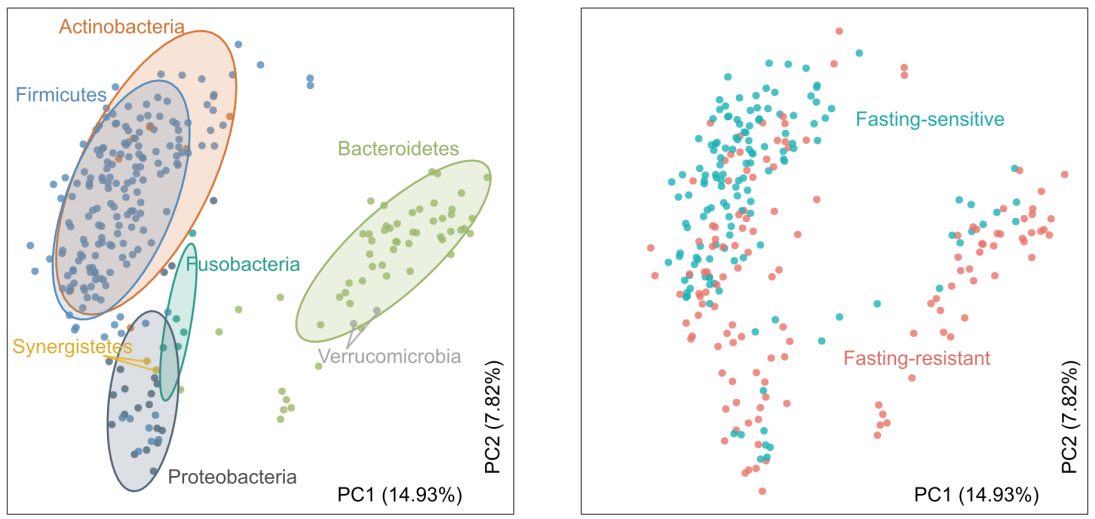


**Supplementary Figure 6| Principal coordinates analysis (PCoA) of the CAZyme profiles of fasting-resistance and fasting-sensitive bacteria.** Bacterial species are shown at the first and second principal coordinates (PC1 and PC2), and the ratio of variance contributed by these two PCs is shown. Species are colored based on their phylogeny at the phylum level (upper panel) or the stratification of FSBs and FRBs (bottom panel). Ellipsoids represent a 95% confidence interval surrounding each phylum.


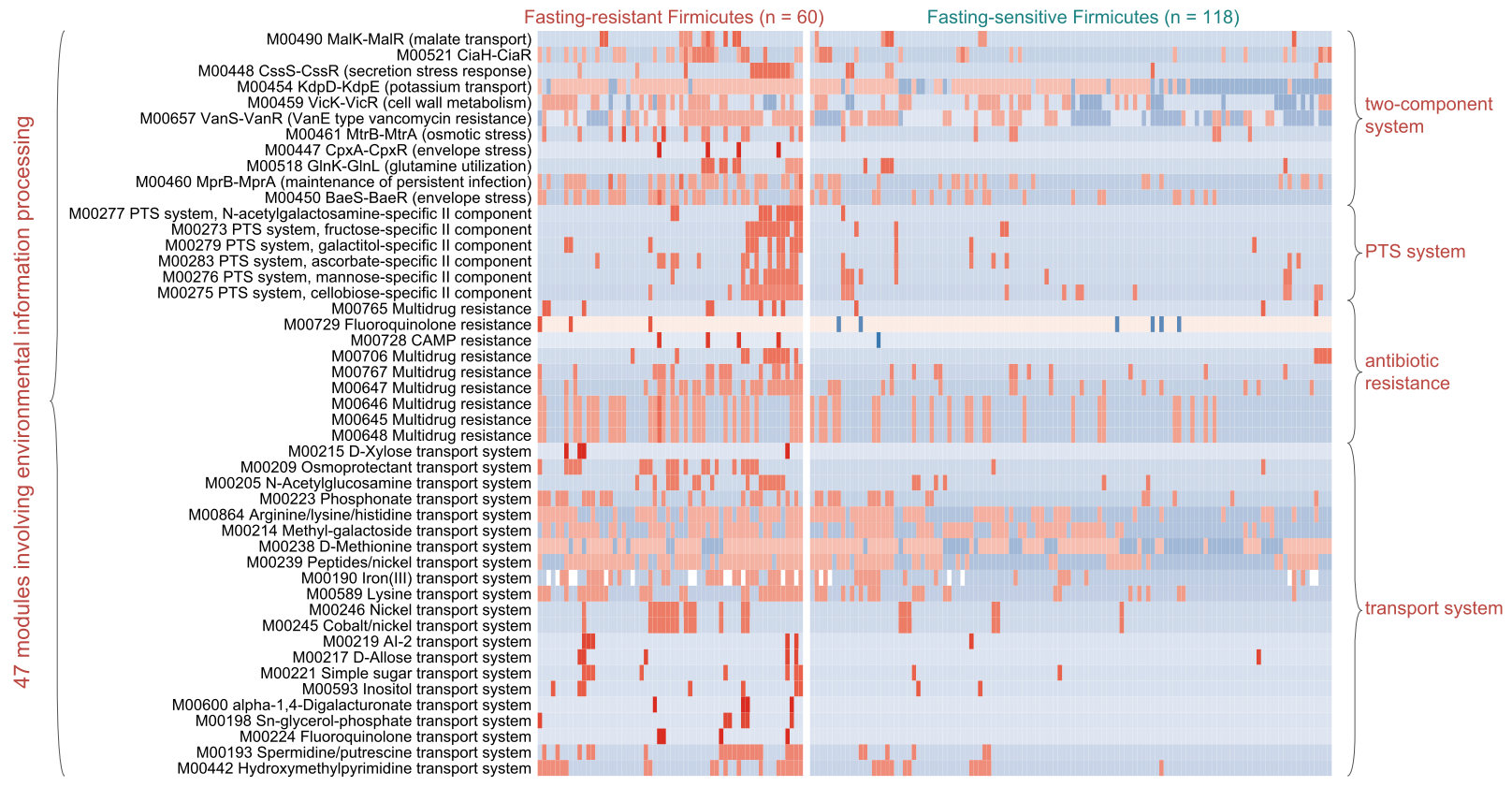


**Supplementary Figure 7| Heatmap showing the abundances of the 47 differential KEGG modules involving environment information processing.** Each row represents a KEGG module, and each column represents a species. The family-level taxonomic assignments of each species are shown in Figure 5c.


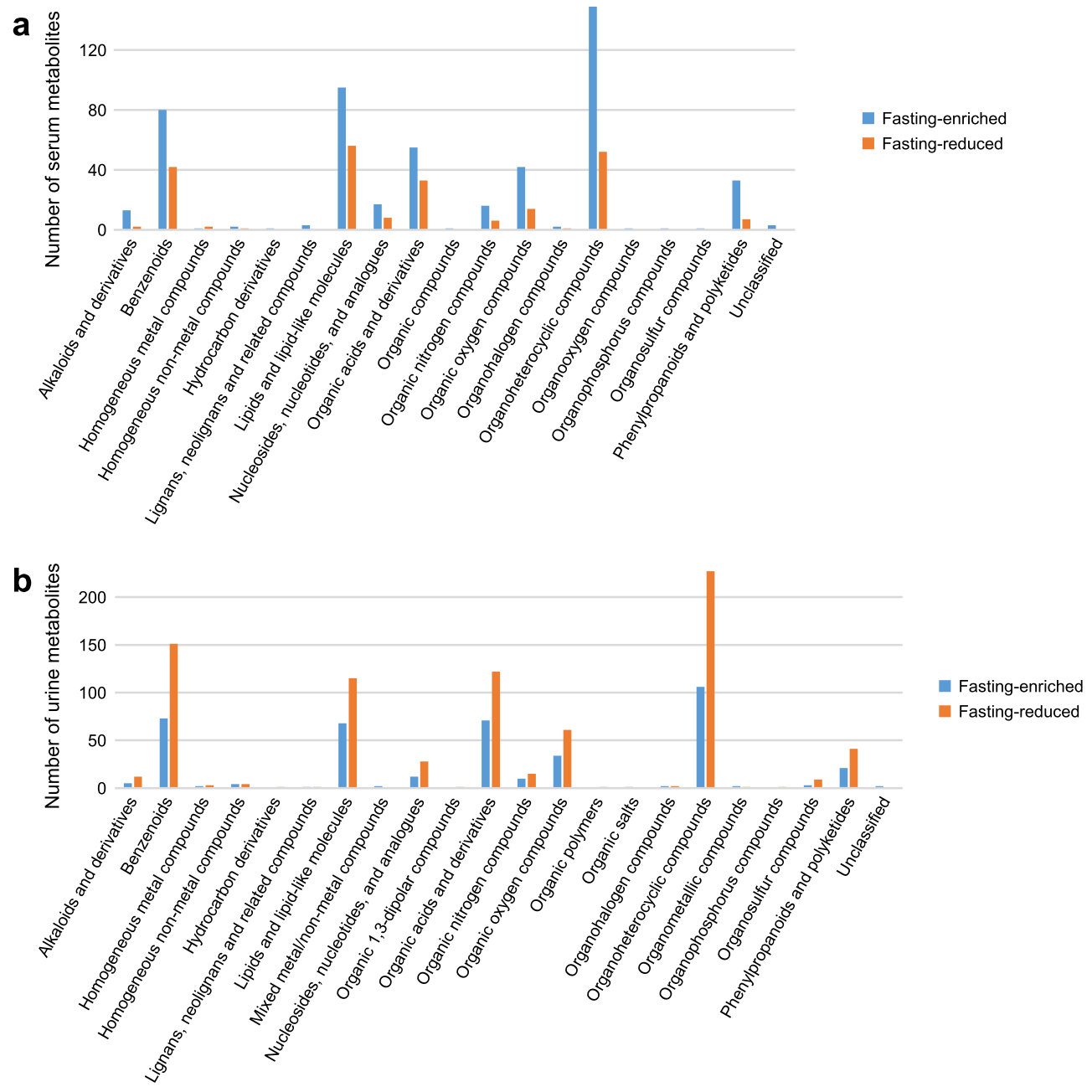


**Supplementary Figure 8| Distribution of serum (a) and urine (b) metabolites that differed in abundance at the T1/T2 timepoints compared with baseline.** Detailed information of these metabolites is shown in Supplementary Table 6-7.

**
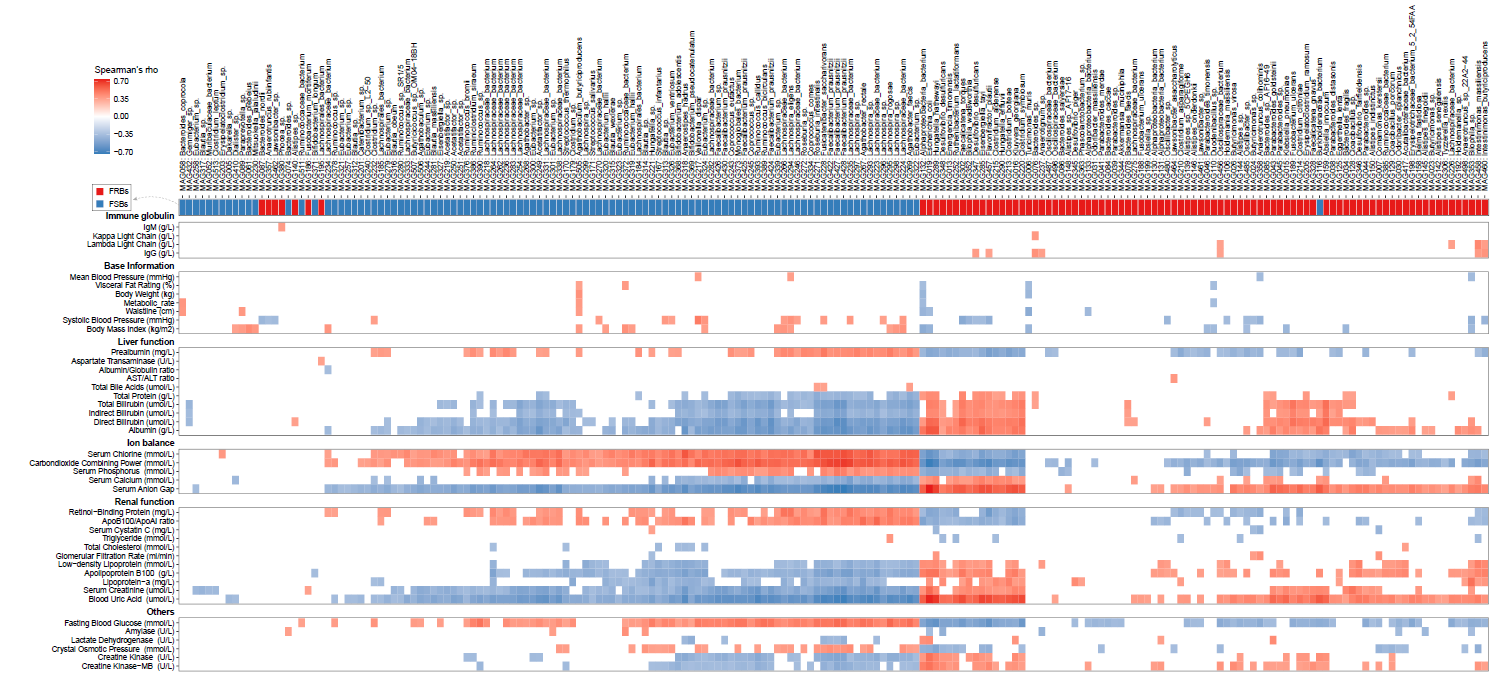
**

**Supplementary Figure 9| The correlation between the fasting-responded species and clinical characteristics.** The colored blocks represent Spearman's |ρ| > 0.35, with a significance level of q < 0.05.
